# 

**Published:** 1898-10-15

**Authors:** 


**Notices of Births, Deaths, and Marriages** are inserted in this column at a charge of 2s. 6d. for an announcement not exceeding four lines.

### NOTICE TO CORRESPONDENTS.

All MS., Letters, Books for Review, and other matters intended for the Editor should be addressed The Editor, "The Hospital," The Hospital Building, 28 & 29, Southampton Street, Strand, W.C.

The Editor cannot undertake to return rejected MS., even when accompanied by stamped directed envelope.

**Notice to Advertisers and Subscribers.**

All Advertisements, Orders for Copies of The Hospital, and Business Communications should be addressed to **THE MANAGER** **(Not to the Editor)**, The Hospital, The Hospital Building, 28 & 29, Southampton Street, Strand, London, W.C.

The Cost of Subscription, post free, is as follows:—Per week, 2½d.; per quarter, 2s. 8d.; per half-year, 5s. 3d.; per annum, 10s. 6d. Foreign:—Per annum, 15s. 2d., payable, in all cases, in advance.

**NOTICE.—Vol. XXIV. of "The Hospital" (April, 1898, to September, 1898), in handsome cloth binding, will shortly be on sale at the Office of this Journal, price 7s. 6d. Cases for binding the Numbers contained in this Volume 1s. 6d. Index to Vol. XXIV., 2½d., post free.**

**The Monthly Part for October is now ready. Price 6d., by post 8d.**

### BOOKS RECEIVED.

"Memorandum and Articles of Association of the East Suburban Medical Protection and Medico-Ethical Society."

Swan Sonnenschein and Co.

"Common Salt." By C. Godfrey Gümpel.

The Women's Institute.

"A Dictionary of Employments Open to Women." By Mrs. Philipps.

Fannin and Co.

"A Synopsis of Surgery." By R. F. Tobin, L.R.C.S.I.

### SCRAPS AND GLEANINGS.

The *Cornhill Magazine* for October has a very amusing article dealing with the humours of hospital life.

It has been decided to abolish street collections in Worcester, and to make instead a house-to-house collection.

The Royal Commission on Sewage Disposal are inspecting the works erected at Exeter for the purification of sewage by the "septic" system.

It is hoped that the new Isolation Hospital at Southampton, the foundation-stone of which was laid the other day, will be finished this time next year.

It is being advocated by certain parties in New York that soldiers in uniform should be allowed to ride free on all railways throughout the country for a period of two years.

The foundation-stone of the extension of the Royal Albert Asylum, Lancaster, for 100 additional young imbecile inmates, was laid on September 28th by the Earl of Derby.

The women students of medicine in St. Petersburg have been ordered to wear a distinctive dress, and not only are the colour and cut specified, but they are bidden to buy the material and to have it made up at a certain establishment.

During the year ended last March there were 664 cases attended at their own homes by the nurses and doctors of the National Maternity Hospital, in Holles Street, Dublin. Of intern cases there were 838 cases treated during the same period.

A Berlin firm has adopted a new idea. They offer to sell music by weight, *i.e.*, so much per kilo for songs, so much for pianoforte music, so much for violin, and so on. Thus are the majority of modern composers in Germany introduced to the public!

The publication of the proceedings at a London police-court the other day, when a poor sempstress was summoned because she could not pay the licence for her dog, her sole companion, and which the magistrate, taking pity on her, allowed her time to pay, has resulted in a sum of close on £100 being sent for this case by the warm-hearted and charitable public.

Despite much objection it has been decided by the Committee of the Cork Hospital Saturday Committee not to discontinue the use of tambourines for collecting purposes, as they considered them the best means of making a successful collection. It has been decided to increase the number of stations from 28 to 38, and to forbear entirely from giving collecting boxes to children: a very right and proper decision.

The Committee of the Bexhill Cottage Hospital scheme are experiencing some difficulty with regard to the erection of the building. The vendor of the land is objecting to what the committee have done—or rather to what they have not done—and threatens drastic measures unless they agree to his proposition to augment the committee, who up to the present have failed to carry out the part of their agreement with him.

The Bristol Royal Infirmary are urgently in need of twelve generous friends to give £1,000 each to their institution. A short time ago an anonymous friend offered to give £100 to the Bristol Royal Infirmary if nine others would give £100 each towards wiping off the debt of £13,000. No response, however, having been made to this offer, the same friend now proposes to give £1,000 if twelve others will each give the same amount, and so wipe off the whole of the debt.

### The Student of Medicine.

### APPOINTMENTS.

H. J. M. Alford, L.R.C.P., M.R.C.S., appointed Resident Dispensary Medical Officer to the Weston-super-Mare Hospital. M. Bailey, M.R.C.S., L.R.C.P., appointed Senior House Surgeon to the Bootle Borough Hospital. A. Boulton, L.R.C.P., M.R.C.S.Eng., D.P.H., appointed Medical Officer of Health to the Horncastle Rural District Council. M. Cahill, L.R.C.P.I. and L.M., L.R.C.S.I. and L.M., appointed Medical Officer of the No. 2 Division of the Felkard Dispensary District. A. Clark, F.R.C.S., appointed Consulting Surgeon to the St. Marylebone Dispensary. M. Connon, M.B., C.M., D.P.H.Aberd., appointed Medical Officer of Health for the Burgh of Montrose. G. F. Cross, M.B., B.Ch., appointed District Medical Officer to the Downham Union. A. N. Davis, L.R.C.P.Edin., L.R.C.S.Edin., appointed Medical Superintendent of the County Asylum, Exminster. A. R. Down, L.R.C.P.Lond., L.S.A., appointed Medical Officer for Bampton. F. N. Grinling, M.R.C.S., L.R.C.P.Lond., appointed Medical Officer of Health to the Walker Urban District Council. H. H. I. Kitchon, L.R.C.P.-Lond., M.R.C.S., appointed Medical Officer of Health for the Heywood Borough. T. C. Jones, L.R.C.P., L.R.C.S.Edin., appointed Medical Officer of Health to the Ruthin Rural District Council. A. K. Reed, L.R.C.P., L.R.C.S.Edin., appointed District Medical Officer to the Taunton Union. D. Whittles, L.D.S.Eng., appointed Dental Surgeon to the General Hospital, Birmingham. F. R. S. Gaman M.R.C.S.Eng., L.R.C.P. Lond, appointed Medical Officer of Health to the Caistor Rural District Council and Medical Officer for the No. 1 District and the Workhouse of the Caistor Union. J. C. Griffiths, M.B., B.Sc.Lond., M.R.C.S., L.R.C.P., appointed Medical Officer and Public Vaccinator for the Tealby District of the Caistor Union. P. M. Kerr, M.B., C.M.Edin., appointed Surgeon to the Dumfries and Galloway Royal Infirmary. J. A. C. Kynoch, M.B., C.M., appointed Medical Officer in the Department of Obstetrics and Gynæcology of the Dundee Royal Infirmary. T. H. Molesworth, M.B., B.C., M.R.C.S., appointed Senior House Surgeon to the Stockport Infirmary. B. L. Paton, B.A., M.B., C.M., appointed Clinical Assistant to the Chelsea Hospital for Women. H. Peck, M.B., appointed Medical Officer of Health to the Chesterfield Urban District Council. Dr. C. Perrott, appointed Medical Officer for the Kingswood, Oldland, and Hanham Abbots District of the Warmley Union. J. H. Ray, M.B.Vict., Ch.M., appointed Medical Officer to the Salford School Board. S. B. Reid, M.A.Cantab., M.R.C.S.Eng., L.R.C.P.Lond., appointed House Surgeon to the East London Hospital for Children, Shadwell, E. H. M. Richards, M.D.Lond., appointed Medical Officer of Health to the Dronfield Urban District Council. R. T. Richmond, B.A.Cantab., M.R.C.S.Eng., L.R.C.P.Lond., appointed Assistant House Surgeon to the Salisbury General Infirmary. P. H. Stratton, M.R.C.S.Eng., L.R.C.P.Lond., appointed House Surgeon to the Children's Hospital, Bradford. P. E. Turner, M.B., B.S.Durh., M.R.C.S., L.R.C.P., appointed House Surgeon to the Kent County Ophthalmic Hospital, Maidstone. G. Wilson, M.D., reappointed Medical Officer of Health to the Meriden Rural District Council.

### THE MEDICAL SCHOOLS.

St. Bartholomew's Hospital and College.—The Entrance Scholarships have been awarded as follows: Junior Scholarship in Chemistry, Physics, and Biology, value £150, C. C. Robinson, J. Burfield, equal. Senior Scholarship in Biology and Physiology, value £75, L. J. Picton. Preliminary Scientific Exhibition in Chemistry, Physics, and Biology, value £50, A. F. Forster. Jeaffreson Exhibition in Latin, Mathematics, &c., T. Jefferson Faulder. Shuter Scholarship in Anatomy, Physiology, and Pharmaceutical Chemistry, value £50, F. C. Shrubsall.

28 & 29, SOUTHAMPTON STREET, STRAND, LONDON, W.C.

### TWO LECTURES

TO THE

### NURSES OF THE LONDON HOSPITAL

BY THE

Hon. SYDNEY HOLLAND.

Price 6d., or 5/- a Dozen.

SECOND EDITION. THIRD THOUSAND.

To be had of Messrs. WHITEHEAD and MORRIS,
9, Fenchurch Street, London, E.C.

